# Tunable In Situ 3D-Printed PVDF-TrFE Piezoelectric Arrays

**DOI:** 10.3390/s21155032

**Published:** 2021-07-24

**Authors:** Alec Ikei, James Wissman, Kaushik Sampath, Gregory Yesner, Syed N. Qadri

**Affiliations:** Naval Research Laboratory, 4555 Overlook Ave SW, Washington, DC 20375, USA; james.wissman@gmail.com (J.W.); kaushik.sampath@nrl.navy.mil (K.S.); gregory.yesner@nrl.navy.mil (G.Y.); noor.qadri@nrl.navy.mil (S.N.Q.)

**Keywords:** PVDF, PVDF-TrFE, 3D printing, smart materials, sensors

## Abstract

In the functional 3D-printing field, poly(vinylidene fluoride-co-trifluoroethylene) (PVDF-TrFE) has been shown to be a more promising choice of material over polyvinylidene fluoride (PVDF), due to its ability to be poled to a high level of piezoelectric performance without a large mechanical strain ratio. In this work, a novel presentation of in situ 3D printing and poling of PVDF-TrFE is shown with a d${}_{33}$ performance of up to 18 pC N${}^{-1}$, more than an order of magnitude larger than previously reported in situ poled polymer piezoelectrics. This finding paves the way forward for pressure sensors with much higher sensitivity and accuracy. In addition, the ability of in situ pole sensors to demonstrate different performance levels is shown in a fully 3D-printed five-element sensor array, accelerating and increasing the design space for complex sensing arrays. The in situ poled sample performance was compared to the performance of samples prepared through an ex situ corona poling process.

## 1. Introduction

Three-dimensional printing of sensors has become increasingly popular as it combines the benefits of additive manufacturing such as rapid prototyping, increased customization, and reduced waste along with the ability to embed sensors directly into a system without additional assembly [1]. This has been successfully demonstrated for various sensing mechanisms ranging from electrochemical detection to resistive strain measurement [2,3,4,5]. However, most current piezoelectric sensors have 2D or 2.5D structures as flat or curved sheets, limiting further geometric modification [6], and additive manufacturing of piezoelectric sensors remains a challenge, in part due to the poling requirements and specialized materials in comparison to capacitive and resistive alternatives. An existing example is the fabrication of a piezoelectric microphone using digital light processing (DLP) and a resin loaded with barium titanate [7].

For 3D-printed piezoelectric sensor fabrication, ceramic piezoelectrics such as lead zirconate titanate (PZT) have been favored due to their unmatched piezoelectric coefficient, d${}_{33}$ = 400 pC N${}^{-1}$ [8]. However, PZT is toxic, brittle, and prone to corrosion. Moreover, the 3D-printing processes involved are complex, expensive, and not widely used. Therefore, PVDF, despite having a substantially lower d${}_{33}$ of 20 pC N${}^{-1}$ [8], has become an increasingly attractive alternative that overcomes all the above-mentioned limitations. It can be printed using fused deposition modeling (FDM), a very inexpensive, widely used 3D-printing technique, which allows for rapid prototyping.

**Figure 1:**
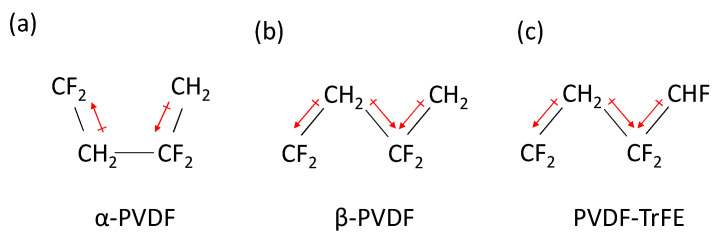
(**a**) α-phase PVDF, (**b**) β-phase PVDF, and (**c**) PVDF-TrFE. The net dipole moment transverse to the polymer chain is seen in the β-phase PVDF and PVDF-TrFE, but not in the α-phase PVDF.

PVDF is a fluorinated hydrocarbon with two hydrogen or fluorine atoms connected to each carbon atom (CF${}_{2}$CH${}_{2}$)${}_{n}$. When cooled from the liquid phase, it forms the nonpolar α phase, as shown in Figure 1. If the fluorine and hydrogen atoms are in the β phase, it creates a dipole moment for each monomer [8]. Mechanical stretching is required to convert the nonpolar α to the polar β phase and to increase the d${}_{33}$ value for use as a piezoelectric sensor [8,9]. By adding barium titanate (BaTiO${}_{3}$) (BTO) particles to PVDF, studies have shown that the β phase content increases significantly [10,11]. A substantially more potent way of achieving the same goal is by using poly(vinylidene fluoride-co-trifluoroethylene) (PVDF-TrFE), a copolymer of PVDF [12]. The additional fluorine atom in the TrFE monomer impedes the formation of the α phase through steric hindrance, causing the material to form the β phase after cooling from the melt [12]. This molecular property of PVDF-TrFE makes it suitable for poling after cooling without further mechanical processing. The average d${}_{33}$ performance of a commercial PVDF-TrFE sensor is −33 pC N${}^{-1}$, which is significantly higher than the −20 pC N${}^{-1}$ reported for PVDF [8]. The ease of creating the β phase content and higher d${}_{33}$ performance makes PVDF-TrFE the superior choice for normal displacement sensors.

**Figure 2:**
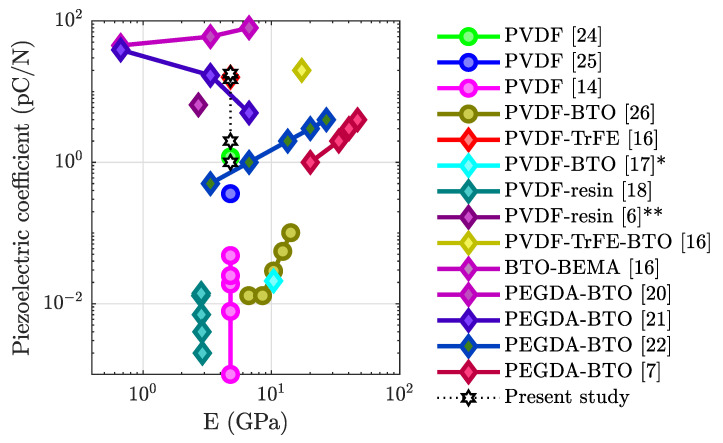
Ashby plot of the piezoelectric coefficient of other 3D-printed polymer piezoelectrics, with the calculated E values based on material filling fractions. Data series represent different publications. Circles are used to mark in situ poling publications, while diamonds are used to mark ex situ poling (unmarked = d${}_{33}$ value, * = d${}_{31}$ value, ** = d${}_{33}$ calculated from g${}_{33}$).

Lee et al. [13] were the first to 3D print and pole PVDF in situ using a high electric field between the print head and the base plate. Dubbed the electric poling-assisted additive manufacturing (EPAM) process, it relies on the resulting shear from printing to transform the melt into the β phase. In Kim et al., PVDF films were 3D printed and corona poling was used to induce the piezoelectric structure [14]. The print was determined to have a d${}_{31}$ value of 4.8 × 10${}^{-2}$ pC N${}^{-1}$, three orders of magnitude smaller than commercial film performance. Ex situ poling of 3D-printed PVDF-TrFE samples has been demonstrated by several groups [15,16] with higher β-phase crystallization, as well as voltages obtained by the samples [15]. In Zhou et al., the d${}_{33}$ performance was seen to be as high as 20 pC N${}^{-1}$ [16]. An Ashby plot, which includes the performance of other 3D-printed polymer piezoelectrics [14,17,18,19,20,21,22], as well as the object of the current study, is shown in Figure 2.

In summary, PVDF-TrFE is a good candidate for 3D-printed piezoelectric devices and has been shown to work well when poled ex situ. However, this two-step approach can be improved. By introducing in situ poling, control over intrasample performance is gained, allowing functional capabilities to also be rapidly prototyped and more exotic behavior to be designed. In this work, we present the ability to print and pole PVDF-TrFE simultaneously, which has not been reported yet to our knowledge. We document the change in the d${}_{33}$ performance as applied electric fields were varied during printing. Through the variation of the applied field, the performance was modulated between elements within a piezoelectric array sample. We characterize and compare these printed samples with conventionally prepared polymer piezoelectric sensors, as well as with samples that were poled ex situ.

## 2. Materials and Methods

### 2.1. PVDF-TrFE Characteristics

The PVDF-TrFE filament used in this study was procured from PolyK Technologies produced with a lab-scale 3D filament extruder. The PVDF-TrFE copolymer powder was purchased from Arkema S.A. containing a mole fraction of 70:30 PVDF to TrFE and a melt flow index of 9.4 kg/10 min at 230 °C and with a 10 kg load. The copolymer powder was thoroughly dried at 100 °C under vacuum overnight. The 3D filament extruder had a screw diameter of 16 mm, and the extrusion temperature was 190 °C. The filament was pulled out of the nozzle at a speed of 1 m/min and cooled with DI water to room temperature.

**Figure 3:**
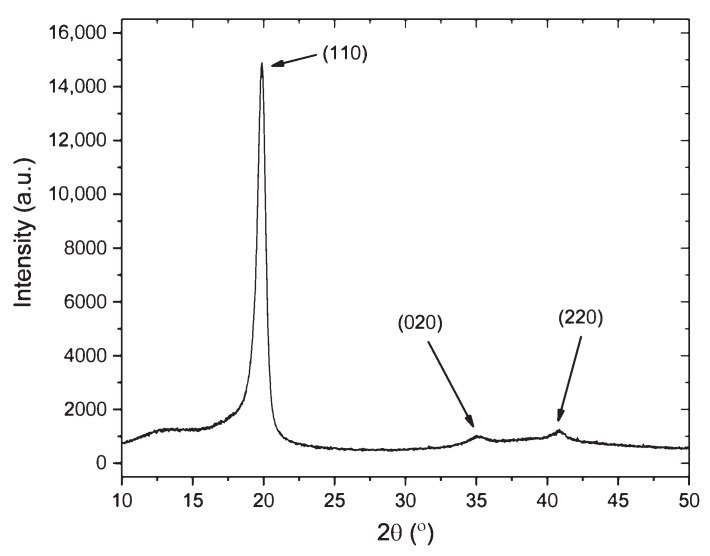
XRD pattern of an in situ-printed and -poled PVDF-TrFE element.

To characterize the crystalline phase information of PVDF-TrFE samples, X-ray diffraction (XRD) measurements were performed using a Rigaku 18 kW X-ray generator producing CuKα radiation from a rotating anode source and a high-resolution diffractometer. PVDF-TrFE elements were cut into 10 mm × 10 mm sections for measurements, and diffraction scans were collected from 10° to 90° 2θ at a rate of 2°/min with a step size of 0.02°. Figure 3 shows the XRD pattern of an in situ-printed and -poled PVDF-TrFE element. The 2θ peaks at 19.8°, 35.25°, and 40.85° correspond to (110), (020), and (220) β-phase crystal reflections [12]. The α-phase reflections at 18.4° and 26.5° were not detected. A crystallite size of 12.48 nm was determined by the Scherrer equation. An 85.8% crystallinity was calculated for the PVDF-TrFE sample by the ratio of the integrated area of the crystalline peaks to the total integrated area. This indicates a high degree of crystallinity, as has been previously reported for PVDF-TrFE compared to PVDF [23].

### 2.2. In Situ Poling Setup

An FDM printer (Ender 3) was used to print the PVDF-TrFE samples, in conjunction with a high-voltage power supply (PS375, Stanford Research Systems). The printer used a 1.75 mm diameter filament and had a nozzle diameter of 0.4 mm. The ground lead of the high-voltage power supply was clipped to the top of the print head to safeguard the interior electronics from an electrical fault. An aluminum plate measuring 5.3 × 3.9 × 0.24′′ served as the high-voltage, positive electrode. A piece of copper tape was attached to the plate, which was clipped to the high-voltage lead of the power supply.

**Figure 4:**
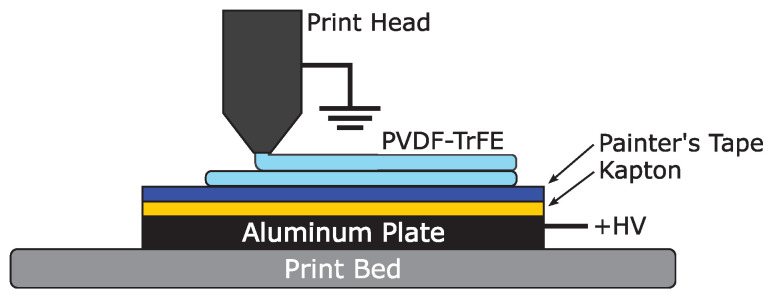
An Ender 3 FDM printer was used to print PVDF-TrFE samples while applying a high electric field. High voltage was applied to the aluminum plate, while the print head was grounded, ensuring the best chance of avoiding an electrical fault in the printer.

The plate was covered in a base layer of Kapton tape and a top layer of painter’s tape. Kapton tape was selected due to its excellent dielectric properties (1 mil thick, rated at 8 kV mil${}^{-1}$), which helped to prevent electrical fault during printing. To also help avoid electrical fault, the voltage was only applied when printing the second layer of the sample. The painter’s tape (5.4 mil thick) helped provide good adhesion between the printed layer and the plate, which was especially important due to the nonstick nature of Kapton tape. It should be noted that, even with the combination of Kapton tape and painter’s tape, samples could not be reliably printed at voltages greater than 8 kV with an initial layer height of 0.15 mm due to dielectric breakdown. A diagram of the setup is shown in Figure 4.

### 2.3. Single Element In-Situ Poling

**Figure 5:**
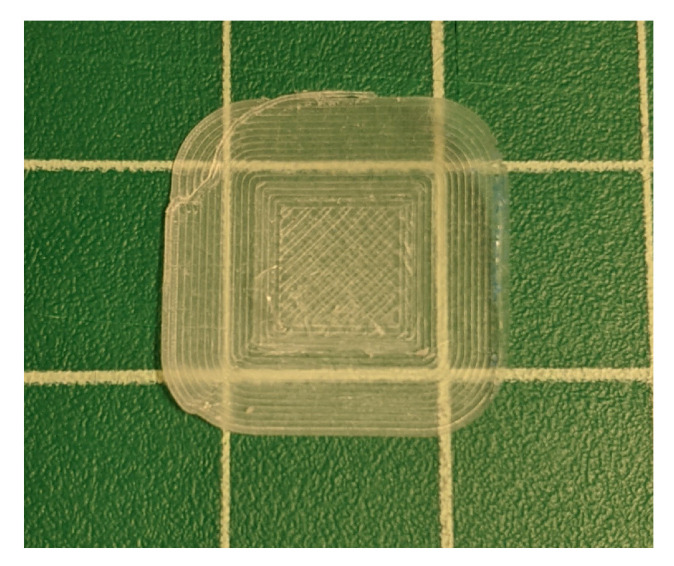
A 3D-printed single-element (10 mm × 10 mm) sample, before electrodes were painted on.

We started with single-element, two-layer prints of PVDF-TrFE, as seen in Figure 5. The CAD model was 1 cm by 1 cm by 0.25 mm with respect to the length, width, and height. The model was sliced in Cura 3.3.1 with the first layer height set to 0.15 mm and the second set to 0.1 mm. The print speed was set to 5 mm s${}^{-1}$. The extruder temperature was set to 210 °C, and the print bed was unheated. Voltage was applied only during the second layer of the print (90 s), to minimize the chance of dielectric breakdown. A sample is shown in Figure 5. The samples were printed at an ambient air temperature of 21–24 °C and between 60 and 65% humidity. Samples were painted on both sides with silver paint from Ted Pella (Product #16062) to serve as electrodes.

The piezoelectric performance was tested in a d${}_{33}$ meter (APC International, Ltd., Product # 90-2030.1) by clamping the sample between the top and bottom probes at its center. The painted samples were placed in the d${}_{33}$ meter, which applied a 110 Hz, 0.25 N sinusoidal force and calculated the d${}_{33}$ value based on the measured electrical output. The low-frequency and applied force safeguarded against nonlinear and triboelectric effects. The d${}_{33}$ meter was calibrated according to the instructions in the manual before measurement, which uses a PZT calibration standard provided with the meter.

### 2.4. Single-Element Ex Situ Corona Poling

**Figure 6:**
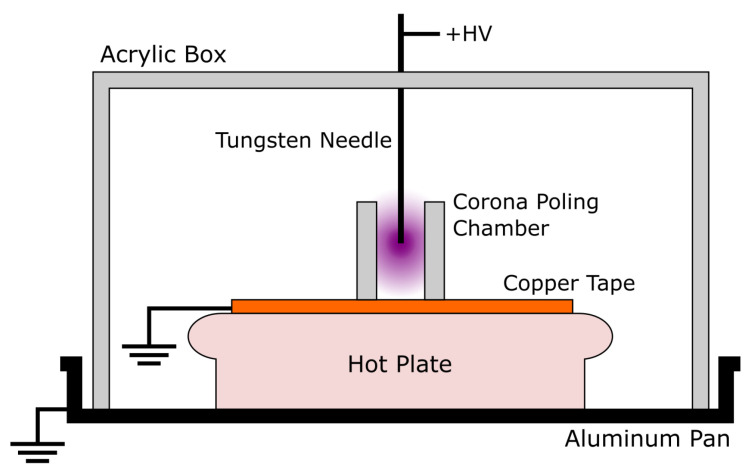
Corona poling setup for PVDF-TrFE. A tungsten needle electrode, brought to a high positive voltage, was used to ionize the air in the poling chamber, which is attracted to the copper ground electrode. The sample was sited on the copper electrode, blocking the charge from reaching it. When combined with the heat from the hot plate, the applied electric field more readily coerces the dipoles in the sample to align with the field to create a piezoelectric device.

To compare the results with conventional poling methods, a corona poling system was used, as shown in Figure 6. The setup included an aluminum pan and a large acrylic box housing providing fail-safe grounding protection. High-voltage wiring was soldered to a tungsten needle (100 μm tip diameter) to ionize the ambient air at the tip of the needle. The ionization voltage required drops with increased curvature, so the use of a sharp needle is recommended. The tip of the needle was housed in a small acrylic box with an open bottom measuring 1 cm${}^{2}$.

The small box was on top of the copper tape, which was connected to the ground lead of the high voltage supply and the aluminum pan. This small box served to contain the charged particles and enhance the density of charge deposited on the sample surface. The voltage applied to the sample depends on its effectiveness as a capacitor. When the electric field gradient around the needle is high enough to ionize air, charged particles are deposited onto the surface of the sample. At some point, affected by the sample’s capacitance, charge leakage between the sample electrodes, as well as that into the setup, a maximum applied voltage is obtained. In addition, when the electric field between the needle and ground electrode is high enough, dielectric breakdown of the air occurs and discharges into the sample. At higher values of applied voltage in this setup, there was a significant chance of discharge occurring, which is why the voltage was not increased further.

The hot plate under the copper tape was used to heat the samples. At high temperatures, thermal energy is enough to randomize the dipole structure of the material. The transition between the ordered state and unordered state occurs at the Curie temperature. Poling is often performed at elevated temperatures below the Curie temperature and then cooled while maintaining the high electric field. Based on a dielectric constant measurement from the manufacturer, we used a Curie value of 118 °C. Here, the ex situ corona poling process was performed on samples above and below the Curie temperature at 145 °C and 80 °C, respectively. The hot plate was brought to the target temperature, then the samples were placed on the hot plate for 1 min before the voltage was applied to the needle. The sample was poled at thus temperature for 30 min. The hot plate was turned off, and the voltage was maintained while the sample passively cooled to room temperature. An IR thermometer was used to verify the maximum set-point temperature during experiments.

Samples were painted with silver paint from Ted Pella (Product #16062), only on the bottom surface (it does not appear to increase the performance of the sample by painting on both sides). When taking out the sample, the fume extractor was turned on to remove the ozone generated by the corona poling process. For this purpose, the fume extractor was equipped with an activated carbon filter (Extract-All, F-987-4A).

### 2.5. Sensor Array

**Figure 7:**
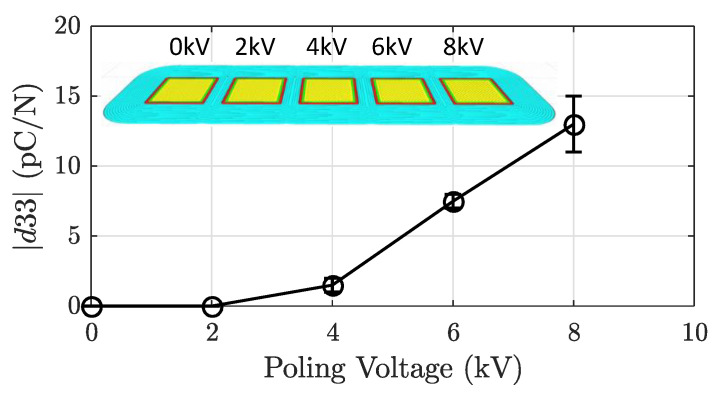
The 3D-printed five-element array, with varying poling voltage for each element. Shown is the average d${}_{33}$ magnitude of the top and bottom sides for each element. The error bars represent the average difference in magnitude between both sides.

A five-element array was printed to demonstrate the potential for in situ poling. By gaining control over the poling voltage applied during printing, the d${}_{33}$ performance can be tuned for each array element. The array was comprised of five of the single-element solid models aligned near each other in Cura. Placing the elements very close to each other caused the brim to overlap and gave a continuous sample, as seen in the Cura tool-path shown in Figure 7.

## 3. Results

### 3.1. Single-Element Performance

The highest d${}_{33}$ performance observed was 18 pC N${}^{-1}$ when simultaneously printed and poled at 8kV. When the voltage applied to the aluminum plate increased, the performance also increased, as can be seen in Table 1. In comparison, we show the performance from ex situ corona poling in Table 2. The electrical field was applied as the sample was brought down from the poling temperature to room temperature. As the temperature applied during poling increased, the d${}_{33}$ performance increased, which was likely due to the increased mobility of the dipoles. The observed performances of the in situ poled samples and the ex situ poled samples show that the in situ poling process is effective and its performance can be modulated within the sample.

It is important to recall that Zhou et al. [16] reported a d${}_{33}$ value of 16 pC N${}^{-1}$ for an ex situ-poled PVDF-TrFE sample, in the same range as the present ex situ and in situ results (Table 1 and Table 2). In their work, the samples were poled in a PE hysteresis loop to obtain the maximum d${}_{33}$ performance. Our ex situ tests were meant to serve as a benchmark to directly compare their work, and the similarity of the results obtained implies that the corona poling setup was sufficient to reach the maximum obtainable performance. The performance of the sample poled while cooling from 145 °C (Table 2) was in fact closer to that reported by Zhou et al. [16], also suggesting that the performance of the sample would not improve with additional poling time. While an in-depth study looking at the effect of poling time and voltage for corona poling is deemed outside the present scope, the results imply that the highest piezoelectric performance was achieved.

### 3.2. Demonstration of a 3D-Printed Sensor Array

In Figure 7, the results of a multi-element printed sample are displayed. By increasing the voltage applied to the aluminum plate during the printing process, the d${}_{33}$ performance increased between the different sections of the sample. The voltage was increased in 2 kV increments between each element, spanning from 0 kV to 8 kV. The d${}_{33}$ results show that the sample was responsive to tuning the poling field strength even with close sample placement.

**Figure 8:**
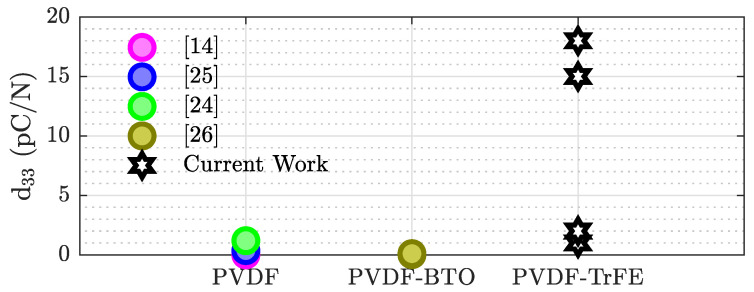
Performance comparison between this work and the literature on in situ-poled piezoelectric polymers. Multiple points for the present work represent the results from different poling voltages shown in Table 1.

As previously mentioned, the reported performance for an ex situ-poled-printed sample was 16 pC N${}^{-1}$ [16] when made of only PVDF-TrFE and 20 and 18 pC N${}^{-1}$ for ones that also contained BTO [10,16]. These values are on the same order of magnitude seen in this work for both ex situ- and in situ-poled samples. This demonstrates that PVDF-TrFE can be effectively poled in situ. In comparison the to reported in situ results, the performances shown here were between one and three orders of magnitude larger than the works cited in Figure 8 [14,24,25,26]. However, none of the in situ results used PVDF-TrFE: they either used PVDF or a combination of PVDF and BTO. The stark difference in performance suggests that there is a low conversion rate between the α and β phase during printing of PVDF. Therefore, our results demonstrate that in situ poling of 3D-printed PVDF-TrFE offers significantly better performance and process efficiency than comparable 3D-printed PVDF.

## 4. Conclusions

In the 3D-printing field, PVDF-TrFE has become a more popular choice of material, due to its ability to be poled to a high level of performance without a large mechanical strain ratio. In this work, we gave a novel presentation of in situ 3D printing and poling of PVDF-TrFE. In addition, the sensor performance was increased from 0 to 15 pC N${}^{-1}$ within a single sample to demonstrate the additional control that in situ poling can provide.

Leading up to this process, ex situ corona poling systems were built and tested on samples of 3D-printed PVDF-TrFE. Even through ex situ poling, PVDF sensors have not shown the same level of performance as in situ PVDF-TrFE sensors. Moreover, the relative ease of the setup makes the in situ poling setup a good solution for future development of fast fabrication, complex, or internally built sensing applications, while its high level of compliance and robustness make it a good candidate for use in soft robotic systems or underwater environments.

In future work, the design space could be expanded by using multimaterial prints, to place conductive layers in between piezoelectric layers to create stacked actuators. Additional work is currently planned to automate the voltage variation within a printed sample, for smoother performance gradients, as well as to increase the height at which in situ printing and poling is possible, to allow for a true 3D print. Further optimization of the current fabrication process could be improved to obtain higher d${}_{33}$ values, which may include the minimization of porosity in the prints or annealing. To transition these sensors from the lab to the field, more material characterization tests should be performed, including the effects of repoling samples, performance decay over time, and other studies that would measure their reliability. 

## Figures and Tables

**Table 1:** Result of the tests performed in situ, for single-element PVDF-TrFE samples. Sample thicknesses were measured with calipers, as they differed slightly from the CAD model.

Voltage (kV)	2	4	6	8
Measured Thickness (mm)	0.19	0.15	0.15	0.16
d${}_{33}$ (pC/N)	1	2	15	18

**Table 2:** A high electric field was applied to samples in a corona poling setup at various temperatures and then cooled while maintaining the field.

Temperature	Unheated	80 °C	145 °C
d${}_{33}$ (pC/N)	4	8	19

## Data Availability

Data sharing not applicable.

## Abbreviations

The following abbreviations are used in this manuscript:

BTO	barium titanate
CAD	computer-aided design
EPAM	electric-poling-assisted additive manufacturing
HV	high voltage
PVDF	polyvinylidene fluoride
PVDF-TrFE	poly(vinylidene fluoride-co-trifluoroethylene)
PZT	lead zirconate titanate
